# Dietetic Management of Adults with Phenylketonuria (PKU) in the UK: A Care Consensus Document

**DOI:** 10.3390/nu14030576

**Published:** 2022-01-28

**Authors:** Louise Robertson, Sarah Adam, Charlotte Ellerton, Suzanne Ford, Melanie Hill, Gemma Randles, Alison Woodall, Carla Young, Anita MacDonald

**Affiliations:** 1 Department of Inherited Metabolic Disorders, University Hospitals Birmingham NHS Foundation Trust, Queen Elizabeth Hospital, Birmingham B15 2TH, UK; 2 NHS Greater Glasgow and Clyde, Royal Hospital for Children, Glasgow G51 4TF, UK; Sarah.Adam@ggc.scot.nhs.uk (S.A.); Carla.Young@ggc.scot.nhs.uk (C.Y.); 3 Charles Dent Metabolic Unit, University College London Hospitals NHS Foundation Trust, National Hospital for Neurology & Neurosurgery, Queen Square, London WC1N 3BG, UK; c.ellerton@nhs.net; 4 North Bristol NHS Trust, Southmead Road, Bristol BS10 5NB, UK; suzanne.ford@nbt.nhs.uk; 5 National Society for Phenylketonuria, Richard House, Winckley Square, Preston PR1 3HP, UK; 6 Adult Inherited Metabolic Disorders Service at Sheffield Teaching Hospitals NHS Foundation Trust, Herries Road, Sheffield S5 7AU, UK; melanie.hill13@nhs.net; 7 Guys and St Thomas NHS Foundation Trust, London SE1 7EU, UK; gemmarandles@gmail.com; 8 Salford Care Organisation, Northern Care Alliance NHS Foundation Trust, Stott Lane, Salford M6 8HD, UK; alison.woodall@nca.nhs.uk; 9 Birmingham Women’s and Children’s Hospital, Steelhouse Lane, Birmingham B4 6NH, UK; Anita.MacDonald@nhs.net

**Keywords:** phenylketonuria, adult phenylketonuria, standard operating procedure, inherited metabolic disorders, dietary management, phenylalanine, protein substitute

## Abstract

There is an increasing number of adults and elderly patients with phenylketonuria (PKU) who are either early, late treated, or untreated. The principal treatment is a phenylalanine-restricted diet. There is no established UK training for dietitians who work with adults within the specialty of Inherited Metabolic Disorders (IMDs), including PKU. To address this, a group of experienced dietitians specializing in IMDs created a standard operating procedure (SOP) on the dietetic management of adults with PKU to promote equity of care in IMD dietetic services and to support service provision across the UK. The group met virtually over a period of 12 months until they reached 100% consensus on the SOP content. Areas of limited evidence included optimal blood phenylalanine reporting times to patients, protein requirements in older adults, management of weight and obesity, and management of disordered eating and eating disorders. The SOP does not include guidance on maternal PKU management. The SOP can be used as a tool for training dietitians new to the specialty and to raise the standard of education and care for patients with PKU in the UK.

## 1. Introduction

Phenylketonuria (PKU) is an autosomal recessive disorder of protein metabolism that is caused by a deficiency of phenylalanine hydroxylase, the enzyme which metabolizes the amino acid phenylalanine to tyrosine. The incidence in the UK is 1 in 10,000 [1], with regional variations. Individuals are recommended to follow a lifelong phenylalanine-restricted diet, supplemented with a low-phenylalanine protein substitute [2,3] to protect the brain from the toxic effect of elevated phenylalanine. In the UK, PKU is detected through neonatal screening, which began in 1969.

Neonatal screening and subsequent early diagnosis and initiation of treatment have changed the outcome of PKU [4], enabling the affected individuals to reach their full cognitive and intellectual potential. The healthcare and social-care savings are highly significant, as individuals do not need institutional care. Those with late-treated PKU are more likely to require special community care packages [5]. The burden of dietary treatment to individuals and carers cannot be underestimated [6,7,8,9].

A range of cognitive sequelae are seen in some patients with PKU [10,11,12]; however, the impact of current phenylalanine levels compared to historical childhood control is still uncertain [3]. There are variations in reported psychosocial outcomes for adults with PKU and indications that partial adherence to treatment negatively impacts on quality of life [8,10].

Dietitians play an important role in helping patients access and achieve effective treatment for PKU. There are several established metabolic centers across the UK that are dedicated to supporting adults living with an inherited metabolic disorder (IMD), including PKU. The needs of adults living with PKU are considerably different from those of pediatric patients, and these change over time as individuals become older. Research has indicated that transition of patients with PKU to adult services is successful with maintenance of metabolic control and high levels of patient engagement [13,14]. Adult clinics also support up to 23% of patients who are not following dietary treatment [15], usually because they maintain phenylalanine levels within target range without treatment (hyperphenylalaninemia) or the dietary treatment was discontinued in childhood by medical teams prior to life-long treatment recommendations. There are adult patients who recognize the benefits of maintaining lower phenylalanine levels but find it too challenging and impractical to sustain dietary treatment. Maintaining contact with this group of patients is important to monitor clinical outcome; to ensure good overall nutritional status; and to keep them informed of any treatment recommendation changes, new research, and developments. A number of adults with PKU choose (and are supported) to restart dietary treatment after a period of discontinuation in adolescence and/or adulthood [16]. Adults with PKU are a highly heterogeneous patient group in terms of treatment history, which includes late diagnosed and late treated, untreated, early treated who have stopped treatment at different stages in childhood, and early and continuously treated patients. This variability in treatment exposure may be reflected in a spectrum of different cognitive, co-morbidities, and life outcomes in adults with PKU attending metabolic clinics.

Dietitians working in the field of adult IMD have scarce access to formal specialty training. Few rotational or dietetic training posts exist within the UK, and therefore identifying the need for and creating a Standard Operating Procedure (SOP) forms part of the standardization of training and dietetic care for adults with PKU. Within the British Inherited Metabolic Disease Group-dietitians’ group, there is a subgroup for adult dietitians. The adult dietitians group meets to specifically discuss dietetic management, develop resources, and arrange adult-focused education and training events to support learning and development within the specialty.

The publication of the first European PKU guidelines in 2017 set out clear standards for care, including for adults with PKU [2,3]. The guidelines explicitly state the need for adult metabolic services that are staffed by healthcare professionals with training in this specialty.

Standard Operating Procedures set out clear guidance about what needs to be achieved to support best practice, ensure transparency, and reduce ambiguity [17]. The aim of this dietetic SOP is to outline the role of the dietetic team in treating adults with PKU. The dietitian is an autonomous practitioner, and this SOP does not replace the dietitian’s decision-making about the care of each individual patient, using evidence and his or her clinical judgment [18]. Dietitians have unique skills to counsel regarding dietary care. This document defines the standards of care that should be offered to all adults with PKU attending specialist care in the UK to ensure equity. This was guided by the first publication of the European PKU guidelines [2,3].

## 2. Materials and Methods

Eight experienced Dietitians specializing in the care of adults with IMDs in the UK met regularly over 12 months (September 2020–September 2021) to discuss the best practice in PKU care in the UK and to create the SOP. The SOP was based on the European PKU guidelines [3] and clinical expertise. Meetings were held virtually for one hour every 1–2 months, with a total of nine over one year. After each meeting, the draft SOP was emailed to all group members who reviewed and commented on this before the next meeting. All ideas and opinions were discussed at the following meting and adjustments made to SOP after 100% verbal consensus at each stage.

This SOP was based on existing SOPs at individual centers which were reviewed and further developed, and then a 100% consensus gained within the group in the meetings. The core group consisted of experienced IMD dietitians working in England and Scotland, and comments were sought from dietitians working in Wales and Northern Ireland to ensure that the whole of the UK was represented.

Once written, the SOP was reviewed by seven adults with PKU via an anonymous online survey, the British Inherited Metabolic Diseases (BIMDG) dietitians’ group, the BIMDG committee, and the National Society for PKU (NSPKU). Feedback was provided and the SOP adapted as required.

The following areas were discussed: (1) glossary, (2) scope, (3) clinical SOP introduction, (4) aims and objectives of the SOP, (5) duties of the adult IMD dietitian, (6) SOP delivery and implementation, and (7) monitoring and assurance. In Appendix A, the section on SOP delivery and implementation examines dietetic assessment and interventions for adults with PKU. These sections include additional adult-specific areas, such as weight management and obesity, eating disorders or disordered eating, and patients who have discontinued dietary treatment.

A separate SOP for maternal PKU will be developed in the future.

## 3. Results

The full SOP is given in Appendix A.

This SOP addresses the standards of dietetic care and intervention for adults with PKU. The aspects of care described in the SOP include the following:

- Aims of dietetic care.
- Dietetic assessment for patient on and off treatment.
- Interventions, including the following:
  - Protein substitutes.
  - Avoidance of foods high in phenylalanine.
  - Prescribed special low-protein foods, e.g., low-protein bread or pasta.
  - Importance of including naturally low-protein foods, such as fruits and vegetables.
  - Specific considerations for females.
  - Adults not on treatment.
  - Those returning to diet.
  - Late treated PKU starting back on diet.
  - Weight management/obesity.
  - Eating disorders.
  - Blood phenylalanine monitoring.
  - Nutritional blood biochemistry/nutritional status.
- Patient follow-up.
- Dietetic contact with patients between outpatient appointments.
- Signposting to other services.
- Discharge or transfer from service.
- Outcome measures.
- Resources.

Variance from the European guidelines [3] occurred where differences in practice across the centers was evident or barriers existed to implementation of the guidelines. Areas requiring further consideration and research included the timescale of informing patients of their phenylalanine blood results, protein requirements, and the inclusion of the assessment and management of disordered eating and eating disorders.

It was agreed that dietitians should report blood phenylalanine results within three days of receipt from the hospital laboratory. All members of the group shared their experience of managing patients with PKU who described disordered eating behaviors. The SOP therefore includes guidance on the identification of disordered eating and eating disorders, provision of support, and signposting to other services if an overt eating disorder was suspected.

It is recommended that the SOP is reviewed every 3 years or is updated within 6 months if any new evidence or guidance is published that necessitates a change in practice. The authors also recommend that all services should perform an annual audit by using a representative sample of patients, using this SOP as a benchmark.

## 4. Discussion

This PKU Adult dietetic SOP is a practical interpretation of the European PKU guidelines [3]. It helps the adult IMD dietitian to translate and further develop the guidance into care in the UK. This document is the first consensus SOP for the dietetic management of an IMD in adults in the UK. Its purpose is to promote care equity for patients with PKU, followed up in IMD dietetic services across the UK and to support service provision. It can be used as a tool for training dietitians new to the specialty.

Patient-centered care is important to build positive dietitian–patient relationships. These relationships enable problem-solving, engagement in care, and earning of patient trust [19]. Working in collaboration with patients and carefully considering their beliefs and values will help guide shared decision-making between the dietitian and the patient [18]. The World Health Organization defines patient-centered care as care that which “meets people’s expectations and respects their wishes” [20]. The dietitian can use the SOP as a treatment guide whilst maintaining patient-centered care at the forefront of management.

To provide holistic nutritional care, the SOP examines aspects of care specific to adults with PKU, including protein intake, weight management and obesity, eating disorders or disordered eating, non-dietary treatment, and patients lost to the service and co-morbidities.

Calculation of protein requirements

The calculation of protein requirements for adults with PKU was considered (Table 1). There are two components: (1) calculation of total protein requirements and (2) calculation of the dose of protein substitute required (which usually provides 52–80% of the total protein intake for a person with PKU treated with a phenylalanine restriction only [21]). The level and type of physical activity undertaken by individuals when calculating their protein requirements should also be considered.

The European PKU guidelines propose “*providing an additional 20% of L-amino acids to compensate for the ‘digestible indispensable amino acid score’ and also a further 20% of L-amino acids to optimize their impact on blood Phenylalanine control*” [3]. The incremental factors serve to compensate for the reduced uptake and utilization of amino acids from protein substitutes and offer metabolic benefits from the large neutral amino acid (LNAA) content. The above refers to protein substitutes derived from L-amino acids, and there may be differences in protein utilization with casein-glycomacropeptide (C-GMP) protein substitutes [22].

Minimum protein requirements are commonly derived from “safe levels” of protein intake [23] that are age-specific until the age of 19 years and then remain constant over the adult lifespan. In a recent review paper, Firman et al. [24] suggests that this may not be suitable for older adults with PKU with higher demands for protein associated with ageing. More research is needed to understand optimal protein needs for adults at different life stages and to investigate the body composition of older adults with PKU.

Given the awareness of overweight and obesity amongst adults with PKU [25], it is recommended that protein requirements be based on ideal body weight [3,26]. It is also important to consider patient tolerance of higher doses of protein substitute and the energy balance implications of additional calories supplied at higher prescribed doses of protein substitute.

**Table 1:** Outlining different ways of calculating protein requirements in adults.

	Parameter	Evidence Supporting	Protein Requirement Recommendations
1	Safe protein intake per kilogram of body weight per day	[23]	0.83 g/kg/day
2	Reference nutrient intake for protein	[26]	0.75 g/kg/day
3	Use of Indicator Amino Acid Oxidation Method for protein requirement calculation	[27]	0.93–1.2 g/kg/day
4	Appropriate protein requirements for older adults (> 65 years)	[26,28]	1.2–1.5 g/kg/day
5	Protein requirements for injury and disease (adults)	[28]	1–1.5 g/kg/day
6	Appropriate protein requirement adjustments for obesity	[3,28]	BMI > 30–75% of calculated requirements for actual body weight BMI > 50–65% of calculated requirements for actual body weight

Weight management and Obesity

In 1982, White et al. [29] observed an increased likelihood of an increased body mass index (BMI) in children with PKU. Since then, several studies have found the female PKU population (both adults and children) to have increased levels of overweight and obesity in comparison to the general population [25,30,31]. In a recent systematic review, Rodrigues et al. [32] conducted a meta-analysis and found that the BMI of patients with PKU was similar to their healthy controls; however, a subgroup of patients with classical PKU had a significantly higher BMI. The meta-analysis dataset included both adults and children; the age range was between 0.2 and 52 years. The authors also noted a trend towards a higher BMI in females with PKU in all studies with male and female datasets.

Interestingly, it has been noted that LDL cholesterol and other biomarkers of increased cardiovascular risk that may be increased in obesity are not elevated in patients with PKU. In fact, studies have shown biomarkers of cardiovascular risk, including LDL cholesterol, were reduced in healthy participants with PKU [33,34]. It is not currently known if the decreased levels of cardiovascular biomarkers in PKU confers a protective effect against cardiovascular events in the PKU population.

The likelihood of a patient with PKU being overweight or obese does not correlate with choice of protein substitute [35] and may be associated with treatment adherence. Cammatta et al. [31] observed no correlation between treatment adherence and prevalence of obesity in Brazilian patients with PKU. However, in UK patients over 16 years old, high phenylalanine levels were found to correlate with obesity [36]. Cammatta et al. [31] also observed that 94% of patients with PKU were sedentary.

It is important that the need for weight-management advice, including advice around exercise and activity, is considered within the dietetic-assessment process for all patients with PKU. Further work is needed to monitor the incidence of overweight and obesity and identify the underlying causes in all patients with PKU. Referral to specialist weight-management services (with appropriate support from the IMD dietitian) may be indicated. Bariatric surgery is also possible for adults with PKU who meet the referral criteria; however, careful consideration is needed for both pre- and post-operative management to ensure that a phenylalanine-restricted diet can be maintained.

Disordered eating and eating disorders

Disordered eating and eating disorders occur in adults with PKU. Disordered eating is described as eating behaviors that are lower in severity and intensity than that of an eating disorder. However, both can have an impact of everyday life of the adult with PKU.

The occurrence of eating disorders is recognized in the European Guidelines for PKU [3], but due to the paucity of the literature, they could only recommend that this area required further study. The prevalence of eating disorders self-reported in the PKU patient population is significantly higher than in the general population [37]. Patients with disordered eating are also at a greater risk of developing eating disorders and should have early referral to specialists in psychology and dietetics [20].

Studies also suggest that patients with poor metabolic control are more likely to exhibit symptoms of disordered eating and may be more at risk of developing eating disorders [21,38]. In adolescents and adults with PKU, the occurrence of eating disorders has not been systematically reviewed and is under-reported, so it may not be detected and treated [3].

Disordered eating patterns may be common in patients with PKU without their having an overt eating disorder; regular health-professional support, especially from a psychologist, may provide some measure of protection [3]. Contact with the patient’s general physician and signposting to local support agencies may be warranted as appropriate.

Diagnosing an eating disorder in a patient with PKU is challenging. Existing validated tools for eating disorders may not be appropriate for in individuals with PKU, as they often answer questions differently, due to their prescribed dietary treatment. This can produce false positive or low sensitivity at identifying an eating disorder [38]. Another challenge is the treatment of PKU versus the treatment of the eating disorder. The treatment of PKU involves a low-protein diet which restricts foods high in protein. This is at odds with the treatment of eating disorders such as anorexia nervosa, where the aim of treatment is to remove the self-imposed restriction of food. Regarding referral and treatment of an overt eating disorder, appropriate national guidelines [39,40] and/or local policies should be followed.

It is important that IMD dietitians support individuals with PKU diagnosed with an eating disorder and work in close liaison with dietitians specializing in eating disorders and the wider MDT in a shared care approach. The eating-disorders team is unlikely to have any experience in managing PKU.

Reporting Blood Phenylalanine Concentrations

The NHS England Specialist Services Quality Dashboard for IMD Services [41] directs laboratories to report results within three days of receipt. The European guidelines [3] advise that the ideal standard for time between blood sampling and receiving results should be no more than five days. Barriers to reporting results within five days of the sample being taken include delays in postal service and samples not being posted/given to the laboratory immediately after the procedure is completed. The Australasian PKU Guidelines do not suggest any specific timeframe but advise that dietitians should report results to patients as soon as possible once received from the laboratory [42]. It is important that blood phenylalanine results are reported promptly so that patients can recall how they managed their PKU in the immediate period prior to the blood test, and timely changes can be advised to maintain metabolic control. The European PKU guidelines also recommend that adults should have their phenylalanine concentrations measured monthly [3]. The current group acknowledged that dietitians can only be responsible for the time between results being reported by laboratories to the patient receiving their results. Therefore, for the purposes of this SOP, a realistic standard for patients receiving their results from the dietitian was agreed at three days from receipt of blood results from the laboratory. The best practice is to report the phenylalanine result as soon as possible, but the group acknowledges that this is not always practical, due to inadequate staffing levels. Three days was agreed on an arbitrary basis and is a pragmatic goal for the timeframe of blood phenylalanine reporting.

Non-Dietary Treatments for PKU

Currently there is only one non-dietary adjunct treatment, sapropterin, that has recently been funded by NHS England only for treating adults with PKU. Dietitians will adjust natural protein and protein substitute intake, as well as (potentially) sapropterin dose, for patients who are responsive to this therapy. In Northern Ireland and Wales, sapropterin is routinely available for people with PKU up to the age of 22, and it is hoped that access will be extended to adults. Scottish healthcare has not yet commissioned sapropterin for routine use as a treatment for PKU. Sapropterin management protocols are currently being agreed.

Maintaining Patient Engagement and Avoidance of Patients Being “Lost to Follow Up”

Adult patients vary greatly in their neurocognitive abilities, from having profound learning disabilities and high levels of dependence on nursing care for engagement with treatment (associated with late treated PKU) to complete independence with the dietary regimen. Adults with PKU can present with levels of functioning in between these points, with subtler executive function deficits.

Patients’ variable neurocognitive abilities or executive function deficits that are associated with their heterogeneous treatment experiences and disorder severity need consideration when organizing adult clinics. Impairment of working memory, planning, cognitive flexibility, and sustained attention [8,10] is likely to impact on consistent clinic attendance.

The European Guidelines recommendation is that all adults with PKU should be under systematic follow-up at specialist metabolic clinics and organization of clinics should support adults’ continued engagement [3]. Mechanisms such as reminders to attend just prior to appointments, additional telephone or text messages prompting attendance, and removal of barriers to re-access clinics after missing appointments support better outcomes than systems which discharge patients after a one- or two-time non-attendance. Transition of patients from pediatric to adult clinics is a point in care when patients might be “lost to follow up” for a variety of reasons. Robust transition arrangements will reduce this [21]. Finally, remote clinic appointments using video or telephone calls may support patient attendance if (independent) travel is a barrier to attending adult clinics.

Shared Care

Services caring for people with long-term conditions need to consider the holistic needs of patients with co-morbidities, particularly if this affects dietary management. Co-morbidities may include diabetes mellitus, cancer, inflammatory bowel disorders, irritable bowel syndrome, and dysphagia (late treated) [43]. Collaboration with other medical teams is necessary to advocate for and support PKU treatment alongside concurrent treatments and management of co-morbidities. Additionally, awareness of the impact of PKU management on concurrent conditions or illnesses is essential to adequately support adults with PKU. Although PKU is not a decompensating metabolic disorder, during any hospital admission, provision of a phenylalanine restricted diet supplemented with a low-phenylalanine protein substitute should be organized and supplied. If there is a requirement for enteral feeding, a modular feed using the protein substitute, a natural protein source, fat and carbohydrate modules, and electrolytes can be designed. IMD dietitians should work collaboratively with services supporting hospital admissions and consider any comorbidities to ensure that the requirements of PKU are considered alongside their treatment.

Monitoring and assurance of the SOP

It is important that the SOP is reviewed regularly (every 3 years) to ensure that it remains up to date and informed by clinical practice. If any new evidence or guidance is published which necessitates a change in practice, the SOP will be revised within 6 months of publication. Adult IMD dietitians can use this SOP as a benchmark to audit their service. By providing agreed and defined national guidance for dietetic treatment of PKU in the UK, this SOP will allow all Adult Inherited Metabolic Disorders (AIMD) services to audit provision of care against an agreed national standard. This will also promote consistency of care between services. The SOP will be disseminated via the BIMDG dietitians’ group. This provides an exciting opportunity for services to collaborate on a national audit or future research, with the SOP defining agreed outcomes of dietetic care.

Limitations

The SOP is based on the consensus opinion drawn from the experience of the authors and their interpretation of a scarce evidence base. As the authors are UK-based dietitians working within the UK National Health Services, there is a primary focus on UK services. Official methodology was not used to reach consensus, but 100% consensus was reached in all aspects of the SOP.

There are minimal outcome data on early and continuous treated adults and late treated adults with PKU. The provision of dietary care within adult IMD services (in the UK) is variable due to lack of funding and limited dietetic staffing, which may prevent the recommendations in the SOP from being incorporated into practice.

This SOP document is for the dietetic care only; it does not include the role of the rest of the IMD team in management of the adult with PKU. As more non-dietary treatments become available and adults are at increased risk of other co-morbidities, e.g., diabetes and metabolic syndrome, then future work on the SOP should include the role of the whole team.

## 5. Conclusions

This is the first dietetic SOP for adults with PKU in the UK. The SOP outlines the role of the dietetic team in treating adults with PKU. The SOP and this supporting publication aim to strengthen service provision and achieve equity in the dietetic management of patients in the UK with PKU. The SOP is a consensus based on experience in an area where there is a limited or minimal evidence base to support dietetic management at the present time.

As further non-dietary treatments are expected to become available in the UK, the SOP will be updated to reflect this. Future work is needed, especially in key areas where current evidence is scarce. These include determining protein requirements across the adult lifespan, developing strategies to effectively prevent and manage obesity, and improving the understanding of etiology and optimal treatment approaches with regard to eating disorders. Research focused on adults with PKU remains a high priority to ensure optimal care throughout the lifespan.

## Figures and Tables

**Figure A1:**
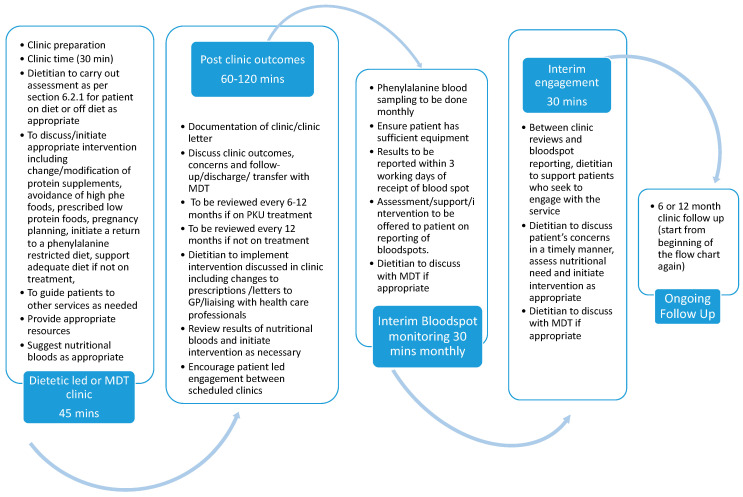
Pathway for Dietetic Management of Adults with Phenylketonuria (PKU) in the UK.

## Data Availability

Not applicable.

## Appendix A

SOP for the Dietetic Management of Adults with Phenylketonuria (PKU) in the UK

Written by Louise Robertson, Sarah Adam, Charlotte Ellerton, Suzanne Ford, Melanie Hill, Gemma Randles, Alison Woodall, and Carla Young on December 2021.

**Contents**

1. Glossary;
2. Scope;
3. Introduction to the clinical SOP;
4. Aims and objectives of this SOP;
5. Duties of the AIMD Dietitian;
6. SOP delivery and implementation;
  6.1 Key stakeholders;
  6.2 Dietetic assessment and interventions for adults with PKU;
    6.2.1 Dietetic Assessment
    6.2.2 Interventions
    6.2.3 Follow up
    6.2.4 Potential outcome measures
    6.2.5 Resources
7. Monitoring and assurance;

**1. Glossary**

- Patient: patient or patient advocate.
- Adult Inherited Metabolic Disorders Dietitian (AIMD dietitian): a dietitian who works with adults who have an inherited metabolic disorder.
- Patient-centered approach: treating the patient as an individual and an equal partner in the healthcare management.
- Psychosocial: how social conditions affect mental health or how someone copes with PKU.
- Neurocognitive: the ability to think and reason. This includes the ability to concentrate, remember things, process information, and understand.
- Capacity: to use and understand information to make and communicate a decision.
- Protein substitutes: a medical food containing all amino acids, except/very small amount of phenylalanine.
- Prescribed low-protein foods: foods manufactured to be very low in protein only found on prescription in the UK.
- Phenylalanine exchange: one exchange is the amount of food that contains 1 g of protein or 50 mg phenylalanine.
- NSPKU: The National Society for Phenylketonuria—patient society in the UK.
- BIMDG: The British Inherited Metabolic Diseases Group—health-professionals interest group in the UK.
- ACBS: Advisory Committee on Borderline Substances—The ACBS is responsible for advising on the prescribing of borderline substances for use in the NHS primary care. Borderline substances are nutritional or dermatological products that have been specially formulated to manage medical conditions.

**2. Scope**

- This SOP outlines the dietitian care pathway for adult patients (16+ years) with Phenylketonuria (PKU) under the care of Adult Inherited Metabolic Disease (AIMD) teams in the UK.
- This SOP does not cover management of maternal and preconception patients with PKU.
- This SOP does not cover the management of acute inpatient admissions
- The role of the AIMD dietitian in PKU care is highlighted.
- This document is to be used with the clinical judgment of the dietitian to tailor it to the adult with PKU.
- The roles of other healthcare professionals are noted, although the entirety of their role in the pathway has not been included.

**3. Introduction to the clinical SOP**

- PKU is the most common inborn error of protein metabolism with an incidence of approximately 1 in 10,000 births, with varying incidence across the UK. Management by restricted dietary intake of phenylalanine (natural protein), along with supplemented phenylalanine-free amino acids (L-AA) or glycomacropeptide (GMP) [3,21], remains the mainstay of management in the UK.
- The current European Phenylketonuria Guidelines [3] recommend that all patients with PKU remain on treatment/restricted diet for life if phenylalanine is >600 µmol/L without treatment.
- Across the UK, there are recognized adult metabolic centers.
- This procedure is necessary to achieve the following:
  ○ Standardize the dietetic care of all adult patients with PKU across the UK.
  ○ Provide a framework to support the dietitian’s decision-making around treatment of patients with PKU.
  ○ Assist development and supervision of AIMD dietitians in the UK and ensure that all AIMD dietitians are providing equal standards of care to patients with PKU.

**4. Aim and Objectives of this SOP**

- To outline the role of the AIMD dietetic team in provision of care to adults with PKU.
- To ensure equity of patient care throughout the UK.
- To agree on standards for patient care to ensure patient safety and optimal care provision by referring to European PKU guidelines 2017 [3].
- To outline the service provision required to provide optimal care.

**5. Duties of the AIMD dietitian**

- It is the responsibility of the AIMD dietetic service to implement the procedures and provide best practice care, as outlined in this document.
- All members of the AIMD dietetic team have a role in advocating for adults with PKU to receive the care that is aligned with this document, unless this is otherwise indicated in the course of the review.
- All members of the AIMD dietetic team are required to escalate any patient management not within the dietetic scope of practice to an appropriate IMD team member.

**6. SOP Delivery and Implementation**

**6.1 Key stakeholders in the SOP**

- The AIMD Dietitians in AIMD centers across the UK.
- The metabolic team, including physician, clinical nurse specialist, and dietetic assistant to support implementation of the SOP.
- Patients, their families, carers and advocates.
- The British Inherited Metabolic Diseases Group (BIMDG).
- National Society for Phenylketonuria (NSPKU).

**6.2 Dietetic Assessment and Interventions for Adults with PKU**

Please also refer to Appendix B Pathway for Dietetic Management of Adults with Phenylketonuria (PKU) in the UK.

**Aims of dietetic care**

- To optimize normal neurocognitive and psychosocial functioning for the patient.
- To support adults with PKU identify their personal aims and goals through the lifecycle.
- To ensure the patient is fully informed on best practice management of PKU in accordance with European PKU Guidelines 2017 [3] and to support the patient to make informed treatment decisions.
- To educate on how to maintain phenylalanine levels between 120 and 600 µmol/L [1].
- To encourage lifelong PKU management.
- To ensure the diet is nutritionally adequate.
- To help promote a healthy weight.

**6.2.1 Dietetic Assessment**

**For patient on a phenylalanine restricted diet**

- Check identification of the patient and seek consent for assessment.
- Medical/surgical history.
- Psychosocial considerations, e.g., change of living circumstances.
- Anthropometry: weight, height, and body mass index.
- Current clinical issues.
- Relevant medications, including protein substitutes and prescribed low-protein foods.
- Biochemistry: nutritional status bloods (refer to European PKU guidelines [3]) and history of blood phenylalanine monitoring.
- Diet history, including the following:
  - Total protein intake (including food sources) and distribution over the day; prescribed and actual intake.
  - Quantity and timing of protein substitute, prescribed and actual intake.
  - How much low-protein food is being used and confidence with incorporating low-protein foods in the diet.
  - Menu planning and cooking skills.
  - Home delivery/local dispensing of protein substitutes and low-protein foods.
  - Discussion regarding patient’s regulation of protein intake, e.g., if he or she is using phenylalanine exchange system/counting grams of protein.
  - Meal timings.
  - Additional vitamin and mineral, omega 3 supplementation, and history of nutritional deficiencies.
  - Overall dietary adequacy, including assessment of total energy intake.
- Patient- and non-patient-related factors affecting treatment management and any specific concerns the patient has relating to his or her PKU.
- Discussion regarding prescription charges (if appropriate).
- To explore relationships with food if concerns are raised.
- Calculated protein requirements; refer to the European PKU guidelines [3].

**For patients not on treatment**

- Check identification of the patient and seek consent for assessment.
- Medical/surgical history.
- Psychosocial issues.
- Anthropometry: weight, height, and body mass index.
- Current clinical issues.
- Medication (including non-prescribed medications, e.g., herbal remedies and probiotics).
- Biochemistry: nutritional bloods (and history of blood phenylalanine monitoring).
- Diet history, including the following:
  - Total protein intake and distribution.
  - Meal timings
  - Any extra nutritional supplementation of vitamins and minerals, trace elements, and omega 3.
  - Overall dietary adequacy.
  - Protein aversion.
- Patient- and non-patient-related factors affecting treatment management and any specific concerns the patient has relating to his or her PKU.
- Exploring barriers to being on treatment.
- Patient education/update on the management of PKU.

**6.2.2 Interventions**

**Protein substitutes**

- Advise on adequate dose.
- Consider nutritional composition of prescribed protein substitute intake; is it nutritionally complete or is additional micronutrient supplementation required?
- Advise on when it should be taken.
- Advise on any new alternative protein substitutes—amino acid/GMP substitutes.
- Offer to arrange patient samples.
- Discuss tolerability and/or barriers to management adherence.

**Patient switching protein substitute or starting new protein substitute**

- Discuss new substitute regimen with patient (if required).
- Send prescription request letter to GP.
- Discuss collection options with patient, e.g., pharmacy or home delivery.
- Seek verbal or written permission to contact home-delivery company to register patient and update the company on the patient’s current prescription if appropriate.
- Advise GP that a home-delivery company will manage prescription requests on behalf of the patient.

**Avoidance of food high in phenylalanine**

- Educate patients about the practicalities of a low-phenylalanine diet, considering individual phenylalanine tolerance and patient preferences.
- Education should include the following:
  - Avoiding high-phenylalanine foods.
  - Suitable natural low-phenylalanine foods.
  - Measuring and counting phenylalanine exchanges.
  - Avoidance of aspartame and discuss suitable phenylalanine-free sweeteners.
  - Appropriate alcohol consumption.
  - Provide sufficient resources to prepare low-phenylalanine meals.
  - Ensure patient understands how to read food labels.

**Prescribed low-protein foods**

- Ensure patients receive enough supplies via ACBS prescription to meet calorie requirements and to allow variety in the diet.
- Advise patients on the availability of new special low-protein foods.
- Provide a list of special low-protein foods available on ACBS prescription.
- Advise on the NSPKU guidance—up to 50 units per month (excluding low-protein milk alternatives and protein substitutes).
- Arrange special low-protein food samples if requested.
- Outline the system on how to obtain regular supply of special low-protein foods on prescription/home delivery, as above.

**Females**

- If appropriate, discuss the importance of the strict low-phenylalanine diet for pregnancy and planning a pregnancy.
- Signpost to obtaining contraception if appropriate.
- Ensure patient knows what to do if she finds out that she is pregnant.

**Adults not on treatment**

- Ensure adequate intakes of macro- and micronutrients.
- Discuss benefits of lifelong PKU management.
- Advise on support available.
- Discuss importance of attending annual appointments and keeping in touch.

**Patients returning to a low-phenylalanine diet**

- An appropriate step-by-step patient-centered approach should be used if a patient wishes to return to dietary treatment.
- Discuss with the patient the responsibilities of the dietitian and the patient.

**Late Treated PKU patients starting back on diet**

Determine:

- If the patient has capacity.
- Baseline behaviors and functions, communication limitations, support needs, and support/care package.
- Possible previous experience of the diet, number of phenylalanine exchanges, and protein substitute used.
- Tolerance of any monitoring, i.e., finger-prick blood taking (including blood spot and capillary).

Identify:

- Number of phenylalanine exchanges, items needed on prescription.
- Key personnel/carers/cooks to teach concepts of low-phenylalanine diet.
- Devise practical menu plans for care homes or equivalent.
- Anthropometric monitoring—weekly weight charts by carers.
- Review dates for carers’ feedback on any behavior changes (improvements).
- 3-month trial to identify if the diet is helping or not.

**Weight Management/Obesity**

- Identify any history of previous strategies used to manage weight or restrict diet. Discuss with the patient the efficacy of these previous strategies from the perspective of weight loss. Explore impact on mental and physical health, and quality of life.
- Assess information on previous attempts at weight loss which were unsuccessful or not sustainable. Use this information to inform the current weight-management strategy.
- Identify health issues or current medical treatment which may impact on the weight-management strategy, e.g., mental-health issues, medical conditions and treatments, socioeconomic factors, age, gender, culture, ethnicity, and personal support mechanisms.
- Assess risk of comorbidities by monitoring lipid profile, blood pressure, and HbA1c (44).
- Assess understanding of the wide range of dietary and nutritional information available. This can be overwhelming and may be a barrier to a weight-management plan.
- Tailor the education and supporting information provided to aid understanding and reduce any barriers which may have formed.
- Individually tailor patient education to help the patient identify realistic goals. This should include no more than two or three diet and lifestyle changes at a time.
- Focus education to promote understanding on food choices to support a low-protein-food diet. For example, at meals, fill half the plate with vegetables, which are naturally low in protein and calories.
- Advise on the lower calorie protein substitute options and also ensure an adequate intake of the protein substitute and micronutrients. This will help ensure nutritional balance which is essential to a healthy weight loss to ensure nutritional to and supporting weight loss.
- Regular physical activity of a moderate intensity is recommended to help support and maintain health and weight loss. NICE (2014) [44] recommends 45 to 60 min exercise, for example brisk walking of cycling, per day as part of a weight loss program.
- If there are significant barriers in place towards weight loss then discuss the possibility of delaying the weight management until a more appropriate time.
- Consider referral or signposting to other services or organizations for support if this is indicated. This could include referring to a weight-management service or program that the AIMD dietitian can then adapt to suit a low-protein diet for patients with PKU.

**Eating Disorders**

- Identify any history of eating disorder from referral into adult service/liaise with pediatric service for more information and if any treatment received or ongoing.
- Identify common behaviors of eating disorders, i.e., missing meals; food avoidance; bingeing behaviors; and compensatory behaviors, including laxative or diet pill misuse, vomiting, or excessive exercise. Identify any issues with body image, irregular meal pattern.
- Identify any physical signs of eating disorders, e.g., excessive tiredness, feeling cold, dizzy, digestive problems, or dental problems unrelated to PKU.
- Identify if not having periods (females) unless due to contraception method or other medical conditions.
- Identity an unusually low or high body mass index (BMI).
- Any rapid weight loss.
- Whether they take part in activities associated with a high risk of eating disorders (for example, professional sport, fashion, dance, or modeling).
- Other mental-health problems.
- If appropriate, educate on effects of low-calorie intake on Phe control/adverse effect on phenylalanine levels.
- Signpost to eating disorders other local resources and charities, e.g., https://www.beateatingdisorders.org.uk/ (accessed on 24 August 2021).
- Refer to appropriate local services, e.g., GP, community mental-health team. Support patient with PKU diet if going through treatment for eating disorder alongside a specialist eating disorders Dietitian.

**Phenylalanine monitoring**

- Review blood phenylalanine control with patient.
- Monthly blood phenylalanine sampling is recommended to support dietetic management and understanding of blood phenylalanine control [1]. Tailored plans can be discussed with patient, e.g., increased frequency, whilst dietary changes are being made.
- Ensure patient has sufficient blood-sampling equipment.
- Support independence with taking blood samples or ensuring an appropriate plan for those unable to take their own blood samples.
- Encourage the patient to take blood samples at the same time of day so that results are comparable, i.e., fasting.
- Clinical nurse specialist can help trouble shoot issues with taking blood samples.
- Agree patient preference to receiving blood phenylalanine results as per local data governance, e.g., telephone call, text, and email.
- Aim to report the blood phenylalanine result back within 3 working days of receipt of the blood result from the lab.

**Nutritional biochemical blood tests**

- Discuss with medical consultant if these are required and refer to the European PKU guidelines [3].

**Advances in research/developments**

- To inform and discuss any new research, treatments, or guidelines as appropriate.

**6.2.3 Follow-up**

- Agree to follow up with patient.
- 6–12 month appointment if on treatment.
- 12 month appointment if not on treatment.
- Less frequent follow-up arrangements may be agreed upon if appropriate (e.g., male patients with hyperphenylalaninaemia).
- The option of video or telephone appointment to be considered if appropriate.

**Follow-up contact in between clinic appointments**

- Contact details provided for queries or help between appointments.
- Encourage patient-led approach to seek support and information as required.

**Guiding patient to access support from other agencies or other Healthcare Professionals (HCP)**

- Referral to other HCPs might be needed, e.g., psychologist.
- Signposting to services outside of the NHS, e.g., IAPT (Improving Access to Psychological Therapies (England)/mental health/GP (Northern Ireland).
- Supporting letters may be needed, e.g., for travel, applying for benefits (for example PIP), employers, etc.

**Discharge/transfer arrangements if appropriate**

- Discharge arrangements will vary between services.
- PKU is a long-term condition which should have lifelong treatment and metabolic-specialist follow-up.
- Some patients may have neurological and cognitive impairments, e.g., poor working memory, meaning that they may need extra support/reminders to attend appointments.
- Dietitians will facilitate patients transfer to another center if they relocate, e.g., university students.

**6.2.4 Potential outcome measures**

- Patient experience feedback.
- Knowledge and skills of managing diet.
- Attending appointments.
- Frequency of blood phenylalanine monitoring.
- Blood phenylalanine concentrations.
- Adherence to protein substitute.
- Variety in diet.
- Healthy body weight.
- No nutritional deficiencies.
- Good quality of life, e.g., PKU Quality of Life Survey.

**6.2.5 Resources**

- NSPKU diet booklet.
- Relevant center resources.
- Picture booklets on the NSPKU website.
- Company literature/websites and recipe books.
- Apps for smart phones or tablets.

**7. Monitoring and Assurance**

SOP group: The SOP working group will review this document every three years to ensure it remains up to date and informed by clinical guidance and evidence. The SOP was written in December 2021, and the review date will be in December 2024. If any new evidence or guidance is published which requires a change in practice, it will be updated within 6 months of publication. The working group will meet to update this.

Service level: AIMD dietetic services should use this SOP as a benchmark to audit provision of services to patients, to highlight gaps in services, and to identify changes in service provision required to conform to the latest guidelines and requirements and the in development of business cases.

It is recommended that each AIMD dietetic service complete an annual audit on a representative sample of patients on key outcomes outlined in this document (such as frequency of consultations and time take to report blood phenylalanine results) and act on the findings of the audit appropriately.

A suggestion for an audit tool which could be used on a representative sample of the patient group is outlined in Appendix C.

## Appendix C

**Table A1:** Suggested Audit tool for the SOP.

Auditing adherence to the SOP for the Dietetic Management of Adults with Phenylketonuria (PKU) in the UK						
Date audit completed:						
Time period covered from:		To:				
Frequency of dietetic clinic consultation (MDT or Dietetic led)						
Patient identifier	Date of most recent clinic appointment offered (D1)	Date of previous clinicappointment offered (D2)	Is the most recent appointment (D1) a rescheduled appointment due to the patient requesting a change of appointment? Y/N	If yes, what was the date of the appointment offered prior to D2 (D3)	Time (weeks) betweenappointments offered to patient (D2–D1 or D3–D1)	Recommended timeframe achieved? Y/N
Frequency of dietetic clinic consultation (MDT- or Dietetic-led)—simplified table						
Patient identifier	D1	D2	Is D1 pt requested reschedule? Y/N	If Y, D3	Time b/w D2–D1 or D3–D1	Recommendation achieved? Y/N?
Time taken to report phenylalanine blood sampling						
Patient identifier	Date result reported by lab (DA)	Date result reported to patient (DB)	Time (days) between date result reported by lab (DA) and date result reported to patient (DB)	Recommendation achieved? Y/N?		

## References

[1] Pandor A., Eastham J., Beverley C., Chilcott J., Paisley S. (2004). Clinical Effectiveness and Cost-Effectiveness of Neonatal Screening for Inborn Errors of Metabolism Using Tandem Mass Spectrometry: A Systematic Review. NIHR Health Technology Assessment Programme: Executive Summaries.

[2] van Spronsen F.J., van Wegberg A.M.J., Ahring K., Bélanger-Quintana A., Blau N., Bosch A.M., Burlina A., Campistol J., Feillet F., Giżewska M. (2017). Key European Guidelines for the Diagnosis and Management of Patients with Phenylketonuria. Lancet Diabetes Endocrinol..

[3] van Wegberg A.M.J., MacDonald A., Ahring K., Bélanger-Quintana A., Blau N., Bosch A.M., Burlina A., Campistol J., Feillet F., Giżewska M. (2017). The Complete European Guidelines on Phenylketonuria: Diagnosis and Treatment. Orphanet J. Rare Dis..

[4] Enns G.M., Koch R., Brumm V., Blakely E., Suter R., Jurecki E. (2010). Suboptimal Outcomes in Patients with PKU Treated Early with Diet Alone: Revisiting the Evidence. Mol. Genet. Metab..

[5] Brown M.C.J., Guest J.F. (1999). Economic Impact of Feeding a Phenylalanine-Restricted Diet to Adults with Previously Untreated Phenylketonuria. J. Intellect. Disabil. Res..

[6] Eijgelshoven I., Demirdas S., Smith T.A., van Loon J.M.T., Latour S., Bosch A.M. (2013). The Time Consuming Nature of Phenylketonuria: A Cross-Sectional Study Investigating Time Burden and Costs of Phenylketonuria in the Netherlands. Mol. Genet. Metab..

[7] MacDonald A., Smith T.A., de Silva S., Alam V., van Loon J.M.T. (2016). The Personal Burden for Caregivers of Children with Phenylketonuria: A Cross-Sectional Study Investigating Time Burden and Costs in the UK. Mol. Genet. Metab. Rep..

[8] Palermo L., MacDonald A., Limback E., Robertson L., Howe S., Geberhiwot T., Romani C. (2020). Emotional Health in Early-Treated Adults with Phenylketonuria (PKU): Relationship with Cognitive Abilities and Blood Phenylalanine. J. Clin. Exp. Neuropsychol..

[9] Rose A.M., Grosse S.D., Garcia S.P., Bach J., Kleyn M., Simon N.-J.E., Prosser L.A. (2019). The Financial and Time Burden Associated with Phenylketonuria Treatment in the United States. Mol. Genet. Metab. Rep..

[10] Aitkenhead L., Krishna G., Ellerton C., Moinuddin M., Matcham J., Shiel L., Hossain S., Kiffin M., Foley J., Skeath R. (2021). Long-Term Cognitive and Psychosocial Outcomes in Adults with Phenylketonuria. J. Inherit. Metab. Dis..

[11] Burlina A.P., Lachmann R.H., Manara R., Cazzorla C., Celato A., van Spronsen F.J., Burlina A. (2019). The Neurological and Psychological Phenotype of Adult Patients with Early-Treated Phenylketonuria: A Systematic Review. J. Inherit. Metab. Dis..

[12] Hofman D.L., Champ C.L., Lawton C.L., Henderson M., Dye L. (2018). A Systematic Review of Cognitive Functioning in Early Treated Adults with Phenylketonuria. Orphanet J. Rare Dis..

[13] Mütze U., Thiele A.G., Baerwald C., Ceglarek U., Kiess W., Beblo S. (2016). Ten Years of Specialized Adult Care for Phenylketonuria—A Single-Centre Experience. Orphanet J. Rare Dis..

[14] Peres M., Almeida M.F., Pinto É.J., Carmona C., Rocha S., Guimas A., Ribeiro R., Martins E., Bandeira A., MacDonald A. (2021). Implementing a Transition Program from Paediatric to Adult Services in Phenylketonuria: Results after Two Years of Follow-up with an Adult Team. Nutrients.

[15] Wood G., Pinto A., Evans S., Daly A., Adams S., Costelloe S., Gribben J., Ellerton C., Emm A., Firman S. (2021). Special Low Protein Foods Prescribed in England for PKU Patients: An Analysis of Prescribing Patterns and Cost. Nutrients.

[16] Ford S., O’Driscoll M., MacDonald A. (2018). Living with Phenylketonuria: Lessons from the PKU Community. Mol. Genet. Metab. Rep..

[17] NHS Creative Guidance for Standard Operating Procedure (SOP) for Management of COVID-19 Risk Assessments. https://www.england.nhs.uk/south-east/wp-content/uploads/sites/45/2021/05/Standard-Operating-Process-for-Management-and-COVID.pdf.

[18] British Dietetic Association Model and Process for Nutrition and Dietetic Practice. The Model and Process. https://www.bda.uk.com/practice-and-education/nutrition-and-dietetic-practice/professional-guidance/model-and-process-for-dietetic-practice.html.

[19] Sladdin I., Ball L., Bull C., Chaboyer W. (2017). Patient-Centred Care to Improve Dietetic Practice: An Integrative Review. J. Hum. Nutr. Diet..

[20] World Health Organization (2015). People-Centred and Integrated Health Services: An Overview of the Evidence. Services Deliver and Safety.

[21] MacDonald A., van Wegberg A.M.J., Ahring K., Beblo S., Bélanger-Quintana A., Burlina A., Campistol J., Coşkun T., Feillet F., Giżewska M. (2020). PKU Dietary Handbook to Accompany PKU Guidelines. Orphanet J. Rare Dis..

[22] Daly A., Evans S., Chahal S., Santra S., Pinto A., Gingell C., Rocha J.C., van Spronsen F., Jackson R., MacDonald A. (2019). The Effect of Glycomacropeptide versus Amino Acids on Phenylalanine and Tyrosine Variability over 24 Hours in Children with PKU: A Randomized Controlled Trial. Nutrients.

[23] Joint WHO/FAO/UNU Expert Consultation (2007). Protein and Amino Acid Requirements in Human Nutrition.

[24] Firman S., Witard O.C., O’Keeffe M., Ramachandran R. (2021). Dietary Protein and Protein Substitute Requirements in Adults with Phenylketonuria: A Review of the Clinical Guidelines. Clin. Nutr..

[25] Gokmen Ozel H., Ahring K., Bélanger-Quintana A., Dokoupil K., Lammardo A.M., Robert M., Rocha J.C., Almeida M.F., van Rijn M., MacDonald A. (2014). Overweight and Obesity in PKU: The Results from 8 Centres in Europe and Turkey. Mol. Genet. Metab. Rep..

[26] Dietary Reference Values for Food Energy and Nutrients for the United Kingdom (1991). Report of the Panel on Dietary Reference Values of the Committee on Medical Aspects of Food Policy. Rep. Health Soc. Subj..

[27] Elango R., Humayun M.A., Ball R.O., Pencharz P.B. (2010). Evidence That Protein Requirements Have Been Significantly Underestimated. Curr. Opin. Clin. Nutr. Metab. Care.

[28] Parenteral and Enteral Nutrition Group (2018). A Specialist Group of the British Dietetic Association. A Pocket Guide to Clinical Nutrition.

[29] White J.E., Kronmal R.A., Acosta P.B. (1982). Excess Weight among Children with Phenylketonuria. J. Am. Coll. Nutr..

[30] Burrage L.C., McConnell J., Haesler R., O’Riordan M.A., Sutton V.R., Kerr D.S., McCandless S.E. (2012). High Prevalence of Overweight and Obesity in Females with Phenylketonuria. Mol. Genet. Metab..

[31] Camatta G.C., de Cássia Kanufre V., Alves M.R.A., Soares R.D.L., de Carvalho Norton R., de Aguiar M.J.B., Starling A.L.P. (2020). Body Fat Percentage in Adolescents with Phenylketonuria and Associated Factors. Mol. Genet. Metab. Rep..

[32] Rodrigues C., Pinto A., Faria A., Teixeira D., van Wegberg A.M.J., Ahring K., Feillet F., Calhau C., MacDonald A., Moreira-Rosário A. (2021). Is the Phenylalanine-Restricted Diet a Risk Factor for Overweight or Obesity in Patients with Phenylketonuria (PKU)? A Systematic Review and Meta-Analysis. Nutrients.

[33] Williams R.A., Hooper A.J., Bell D.A., Mamotte C.D.S., Burnett J.R. (2015). Plasma Cholesterol in Adults with Phenylketonuria. Pathology.

[34] Mazzola P.N., Teixeira B.C., Schirmbeck G.H., Reischak-Oliveira A., Derks T.G.J., van Spronsen F.J., Dutra-Filho C.S., Schwartz I.V.D. (2015). Acute Exercise in Treated Phenylketonuria Patients: Physical Activity and Biochemical Response. Mol. Genet. Metab. Rep..

[35] Daly A., Evans S., Pinto A., Jackson R., Ashmore C., Rocha J.C., MacDonald A. (2020). The Impact of the Use of Glycomacropeptide on Satiety and Dietary Intake in Phenylketonuria. Nutrients.

[36] Robertson L.V., McStravick N., Ripley S., Weetch E., Donald S., Adam S., Micciche A., Boocock S., MacDonald A. (2013). Body Mass Index in Adult Patients with Diet-Treated Phenylketonuria. J. Hum. Nutr. Diet..

[37] Cazzorla C., Bensi G., Biasucci G., Leuzzi V., Manti F., Musumeci A., Papadia F., Stoppioni V., Tummolo A., Vendemiale M. (2018). Living with Phenylketonuria in Adulthood: The PKU ATTITUDE Study. Mol. Genet. Metab. Rep..

[38] Luu S., Breunig T., Drilias N., Kuhl A., Scott Schwoerer J., Cody P. (2020). A Survey of Eating Attitudes and Behaviors in Adolescents and Adults with Phenylalanine Hydroxylase Deficiency. WMJ.

[39] NICE 2020 (2020). Eating Disorders: Recognition and Treatment NICE Guideline NG69. https://www.nice.org.uk/guidance/ng69.

[40] SIGN Evidence-Based Guidelines (2021). Eating Disorders, A National Clinical Guideline, Consultation Draft May 2021. https://www.sign.ac.uk/media/1849/20210511-ed-draft-v115-peer-review.pdf.

[41] NHS England (2020). Highly Specialised Services Quality Dashboards—Women and Children Metric Definitions for 2020/21. https://www.england.nhs.uk/publication/highly-specialised-services-quality-dashboards-women-and-children-metric-definitions-for-2020-21.

[42] Inwood A.C., Balasubramaniam S., Wiley V., Kreis C., Harrigan K. Australasian Consensus Guidelines for the Management of Phenylketonuria (PKU) throughout the Lifespan. https://www.mdda.org.au/wp-content/uploads/2017/10/Australasian-PKU-Lifespan-Guidelines-FINAL-Endorsed-5.10.2017.pdf.

[43] MacDonald A., Ahring K., Almeida M.F., Belanger-Quintana A., Blau N., Burlina A., Cleary M., Coskum T., Dokoupil K., Evans S. (2015). The Challenges of Managing Coexistent Disorders with Phenylketonuria: 30 Cases. Mol. Genet. Metab..

[44] Obesity: Identification, Assessment and Management Clinical Guideline. https://www.nice.org.uk/guidance/cg189/resources/obesity-identification-assessment-and-management-pdf-35109821097925.

